# Insights on my future job: implementing near-peer shadowing program for operating room freshmen

**DOI:** 10.1186/s12909-021-03071-2

**Published:** 2022-01-30

**Authors:** Mahboobeh Khabaz Mafinejad, Hamed Sarani, Azadeh Sayarifard, Daryoush Rostami, Fatemeh Shahbazi, Larry Gruppen

**Affiliations:** 1 Health Professions Education Research Center, Education Development Center, Department of Medical Education, Tehran University of Medical Sciences, Tehran, Iran; 2 Pregnancy Health Research Center, Zahedan University of Medical Sciences, Zahedan, IR Iran; 3 Center for Academic and Health Policy, Tehran University of Medical Sciences, Tehran, Iran; 4 Department of Anesthesiology, Faculty of Paramedicine, Zabol University of Medical Sciences, Zabol, Iran; 5 Department of Community Medicine, School of Medicine Zahedan University of Medical Sciences, Dr. Hesabi Square - Medical Sciences Campus, Zahedan, Iran; 6 Education Development Center, Zahedan University of Medical Sciences, Zahedan, Iran; 7 Department of Learning Health Sciences, University of Michigan, Ann Arbor, MI USA

**Keywords:** Operating room, Early exposure, Shadowing, Job motivation, Job diagnostic

## Abstract

**Background:**

As a main challenge in paramedical faculties of medical sciences, freshmen lose interest in their academic field of study and then job motivation. Lack of developed knowledge about their academic field and unfamiliarity with their future job’s tasks and roles contribute to freshmen’s job motivation loss. Various interventional programs have been implemented to improve students’ job motivation by familiarizing them with their future job’s duties and responsibilities.

**Methods:**

This was one-group pretest-posttest design study in 2019–2020. Students grouped into pairs of a freshman (shadowee) with a senior (shadower) in a clinical setting during shadowing program. This program helps freshmen to comprehend and discover realities of their academic field and can help them change their perspectives regarding their future job’s duties and responsibilities. The shadowees’ main task was reflective observation on operating room events and interactions and to be active in the program, several tasks e.g., how to wear gloves, guns, and disinfect equipment were assigned to them exclusively under the supervision of senior students. The Hackman and Oldham’s Job Diagnostic Survey (JDS) questionnaire and a novel Job Motivation Survey (JMS) questionnaire were distributed among participants.

**Results:**

Fifty freshmen majoring in operating room participated in the shadowing program from November 2019 to January 2020. Before and after the program, they completed Hackman and Oldham’s job diagnostic survey and researcher-made job motivation survey questionnaires. Results were indicative of a significant difference in job diagnostic survey questionnaire score, where overall pre-test and post-test scores before and after the intervention were 57.78 (±9.78) and 68.58 (±5.02), respectively; the score difference was statistically significant (*P* < 0.001). Moreover, the overall pre-test and post-test scores of the job motivation survey questionnaire were 25.16 (± 9.75) and 39.80 (±5.18), respectively; this score difference was statistically significant (*P* < 0.001).

**Conclusion:**

Shadowing program improved freshmen’s realistic perception of their future job’s duties and responsibility, and hence enhancing their job motivation and job recognition. As future work, in various disciplines, further studies need to evaluate the impact of such interventional programs in providing early insights for freshmen as well as in providing guidance on their plans for education, and future job.

## Background

Clinical education phase plays a major role in developing professional competencies in student majoring in paramedical field, including Operating Room (OR) [1]. The OR undergraduate program is a branch of health sciences in which students are familiar with the principles of the operations and new surgical technologies in specialized and sub-specialized surgeries, and learn to care for and assist patient management before, during and after a surgery. OR technician is a distinct professional pathway from nursing. Graduates of OR play a role as a member of the surgical team to help perform a surgery with desirable results in hospital operating rooms, diagnostic-therapeutic intervention and care centers.

Studies show that a large gap exists between preclinical and clinical training for OR students [2, 3]. Current curricula do not provide students with opportunities to acquire practical skills, and hence forcing them to face challenges later in their clinical phase [4]. In clinical phase, students confront changes in learning and teaching styles [5], which may increase their stress [6, 7]. Furthermore, while facing new situations, tasks, duties, and responsibilities in OR setting, students may confront anxiety and unpleasant experiences that may reduce their motivation to future job [8]. Although the integration of preclinical and clinical phases is taking place in most universities around the world, the integration in the paramedical disciplines has not been considered and the preclinical and clinical phases are two separate stages with long time intervals in Iran. On the other hand, operating room students enter the clinical environment after three semesters from the beginning of curriculum in Iran. In fact, after a year and a half of the pre-clinical phase, students can apply the topics learned in theoretical courses in a practical way, which can reduce their motivation. Despite the revision of the undergraduate curriculum in the operating room by reducing the theoretical courses and upgrading the internship program, but it seems that providing opportunities for effective clinical encounters are not well considered [9], and these deficiencies can affect students’ motivation to learn and acquire knowledge and skills.

According to previous studies, several factors may affect students’ perspectives regarding their future profession; changes in educational environment, different teaching methods in clinical settings, learning in unpredictable situations, and lack of knowledge regarding their future profession’s duties and responsibilities in clinical environment [10–12]. Vanichnatee et al., emphasized that students may lose interest in their academic field and motivation to their future profession due to low job recognition and inadequate understanding of the gap between theoretical knowledge and clinical practice [13]. Furthermore, Kass et al. mentioned that lack of job diagnostic is one of the most common complaints among university students, with studies suggesting its link to poor grades, drop out, and behavioral problems [14]. Despite the revision of the undergraduate curriculum in the operating room by reducing the theory courses and upgrading the internship program, but it seems that providing opportunities for effective clinical encounters are not well considered [15], and these deficiencies can affect students’ motivation to learn and acquire knowledge and skills. Operating room students should be brought up in a clinical environment due to the clinical nature of the field, makes it more important to strengthen the clinical skills of these students as much as possible in their future careers to perform better [16]. According to Mafinejad et al., from the earliest stages of training, acquiring required skills to work in a real clinical environment is crucial [17]. Furthermore, based on the educational needs of paramedical students during their study, it is necessary to use various educational interventions to strengthen the learning process and motivate the field of study [18]. Nowadays, health education system across the world now emphasise early clinical exposure program towards horizontal and vertical integration and contextual learning [19].

Shadowing programs has been implemented by reputable medical education centers to provide early clinical experiences for students and to increase their participation in learning process [20, 21]. The shadowing program exposes learners to real-world work situations using a coherent and particular plan. Freshmen accompany seniors during their presence in the clinical settings to learn and identify prospective job’s duties and responsibilities, work processes and challenges, professional relationships, workplace characteristics, interactions and communications among healthcare team members, and prospective job’s challenges [22]. In Iran, the common model of clinical education for students is under the supervision of faculty members. A review of studies shows that education time constraints, diverse and overloaded duties, uncertainties regarding the clinical responsibilities of clinical teachers, lack of financial support, and priority of research over education are the most important obstacles affecting clinical teaching under the supervision of faculty members [23, 24]. Although this model is considered desirable due to the supervision of faculty members, but in the opinion of faculty members, students and professions, the quality of clinical education have been evaluated as inadequate. Therefore, the need to conduct a program which uses new teaching methods is considered [25]. The use of experienced and efficient senior students as peers in educating students has opened a new horizon in clinical education [26, 27].

In most shadowing program, by linking pre-clinical students with near-peers, prepares them for their future job and changes their job perspective [8]. During this program, seniors (shadowers), share their consecutive years of teaching and learning experience in clinical settings with freshmen (shadowees) [28]. The advantages of this program are as follows: (*i*) enhancing the participatory learning climate; (*ii*) empowering senior students to teach; (*iii*) improving communication skills among students of different levels; (*v*) consolidating the rudimentary knowledge of pre-clinical students; and, (*vi*) application of theoretical knowledge in practice [28, 29]. According to previous studies, a major outcome of such programs is reducing learners’ stress and anxiety through educational and psychological supporting approaches, which in turn will enhance their job motivation [30, 31]. However, Wang et al. suggested that further studies are required to investigate the effect of the shadowing program on freshmen’s job motivation in disciplines other than medicine [22].

To the best of our knowledge, shadowing programs have not yet been implemented for OR students; and hence, this study evaluates a shadowing program for OR students, examining whether this program can increase OR freshmen’s job diagnostic and job motivation. Previous studies have not used valid and reliable tools to measure the impact of this program on student’s future job diagnostic and job motivation, nor have they focused on the implementation process of this program as well as its effectiveness from participants’ perspectives [32, 33]. Consequently, this study aimed to assess the effect of near-peer shadowing program on job diagnostic and job motivation of new OR students.

## Methods

This was one-group pretest-posttest design study in 2019–2020 on paramedical faculty’s OR students. After receiving approval for the research plan and code of ethics (IR.ZUMS.REC.1398.300) from Zahedan University of Medical Sciences, samples were taken from OR freshmen using convenience sampling. Informed consent of the participants was obtained. Probationary students and any from other universities were excluded from this study.

### Setting

The study was carried out in Zabol and Zahedan Universities of Medical Sciences, Iran. OR curriculum has been delivered as a traditional Flexnerian 4-year undergraduate bachelor program. The undergraduate bachelor of OR curriculum in Iran is divided into two phases; including preclinical (three semesters) and clinical phases (five semesters). Preclinical phase of curriculum has been based on the model of teaching that engage students in classrooms setting. In the clinical phase, OR students are exposed to the operating room environment, during which they become acquainted with the realities of the chosen major in the clinical environment and apply theory in practice.

### Implementation of shadowing program

A multi-aspect evaluation of the shadowing program was performed before, during and after the program implantation.

#### Before implementation

To select clinical students (shadowers), the Hackman and Oldham’s Job Diagnostic Survey (JDS) questionnaire and a novel Job Motivation Survey (JMS) questionnaire, designed for this study, were distributed among juniors and seniors. Moreover, to select appropriate shadowers, total GPAs of 36 candidate students were inquired from the university. Fourteen out of thirty-six students with the highest JDS scores and total GPA were selected as shadowers, and an orientation session was held for them. In this session, the shadowers were introduced to the program and were informed about the program’s goals, process, and tasks. In this session, on how to introduce operating room tools, equipment, and different parts of the operating room, to present the duties of operating room staffs, to create an attitude towards the job to students and encourage them to rethink the expected professional roles was emphasized. After completing the program, a cooperation certificate and a gift card were awarded as incentives to the shadowers.

This program was as voluntarily part of the educational course for OR freshmen as shadowee. Shadowee were newcomers to the undergraduate operating room curriculum. Before the program, the shadowees were required to complete JDS and JM questionnaires. Then, for the shadowees, briefing sessions on the following were held: (i) professionalism and etiquette in OR; (*ii*) introductions of OR team members; and, (*iii*) the need to communicate effectively with other OR team members. The shadowees were requested to refrain from any involvement in patient treatment and were only allowed to observe the clinical environment reflectively as well as identify upperclassmen’s role and tasks in OR carefully. After the sessions, the coordinator randomly assigned a shadowee to a shadower while considering schedule compatibility in clinical rotations. To coordinate the presence of students in OR environment, necessary moral and legal approvals were formerly obtained, and administrative correspondence was formally established between the teaching hospitals and the university.

#### During implementation

The shadowing program was implemented for freshmen in a minimum of two working days. The duration of the shadowing program was 8 to 10 h for each shadowee according to the conditions of the operating room clinical environment. To prevent interference in the workload of shadowers and staffs, the hours allotted to each shadowee were allocated during the week according to the conditions of the operating room on different days of the week. Matching was done randomly and one-to-one. At the beginning of the program, the coordinator presented the key points to the shadowees, who were then paired with the shadowers, one to one. The shadowers and shadowees were distributed to the designated teaching hospitals, each having at least six parallel ORs. In intervals of students’ presence in the program, the coordinator rotationally visited different ORs to follow up and resolve possible challenges. Furthermore, according to the program guideline, the shadowees’ main task was reflective observation on OR events and interactions and to identify the upperclassmen’s duties and responsibilities. In order to the shadowees to be active in the program, several tasks (e.g., how to wear gloves in OR, wear guns, and disinfect equipment) were assigned to them exclusively under the supervision of senior students when not interfering with their duties and those of other OR team members. At the end of each encounter with operating room, shadowee can discuss with his/her shadower on their observations and reflections of the experience in reflection time. Furthermore, coordinator meet with students in two smaller groups for reflection. This component was added to the program because there was enough time for discussion about the shadowees’ experiences. Coordinator of the program guide the session by posing reflective questions addressing feelings, experiences, and views of shadowees.

#### After implementation

One week after the end of the program, JDS and JMS questionnaires were manually distributed and filled by the shadowees. If the completed JDS questionnaires were not received, reminders were sent a week later to collect them all.

### Assessment tools

#### Job Diagnostic Survey (JDS) questionnaire

In this study, a 15-item shortened version of Hackman and Oldham’s JDS questionnaire was used [34]. Hackman and Oldham JDS not only use for people who have been worked in a given field, but also for university students’ job motivation and diagnostic across on different degrees and academic years in different studies [14, 35]. Validity and reliability of Persian version of JDS has been confirmed in several studies [36, 37]. In addition, two questions from the full version of the Hackman and Oldham’s questionnaire were added to investigate the role of feedback in the profession and communication with OR colleagues considering the interactive nature of OR. Cronbach’s alpha of the 17-item questionnaire was 0.78. The items of our final questionnaire were from different dimensions of the JDS questionnaire: skill variety, task identity, task significance, autonomy, feedback from the job itself, feedback from agents and exposure to colleagues. The response to each item of this questionnaire was scored on a five-point Likert (very/mostly accurate, slightly accurate, uncertain, slightly inaccurate, and very/mostly inaccurate). Items 3, 6, 9, 12 and 15 of the questionnaire were inverted in scoring.

#### Job Motivation Survey (JMS) questionnaire

A novel questionnaire was designed for this study to examine OR students’ motivation to pursue profession related to their academic field of study. This questionnaire consisted of five general questions rated on a scale from completely agree (9) to completely disagree (1). In order to evaluate the questionnaire’s psychometric properties, we checked content-related evidence for validity (checking the necessity and clarity of questions, removing all ambiguities and other common item errors, reviewing the cultural sensitivity of items) by 10 experts qualitatively [38]. Experts discussed the comprehensibility and clarity of all items. Cronbach’s alpha of the questionnaire was 0.76.

### Data analysis

Statistical analysis was performed using SPSS software (version 26) [39]. Paired-t-test was used to compare the before-intervention (pre-test) and after-intervention (post-test) results of job diagnostic and job motivation survey questionnaires per item and in total. The significance level was considered to be less than *p* = 0.05. Respectively, Effect Size (ES) above 0.8, 0.5, and 0.2% was indicative of strong, moderate, and weak effectiveness of this program.

## Results

Fifty freshmen participated with the mean age of 19.7 years and standard deviation of 1.91. Two out of 52 freshmen candidates were guest students, and hence removed (per exclusion and inclusion criteria). Out of 50 participants, there were 27 (54%) female and 23 (45%) male. Before and after of the program, total pre-test score of job motivation survey questionnaire was a mean of 25.1 with 9.75 standard deviation, whereas the mean of the total post-test score of job motivation survey questionnaire was 39.8 (5.18 standard deviation). OR freshmen’s job motivation significantly increased considering pre-test and post-test scores of job motivation survey questionnaire (*P* < 0.001). In all items of the job motivation survey questionnaire, the intervention program was strongly effective (ES ≥ 0.8) (Table 1).

**Table 1:** Mean and standard deviation of JMS items before and after the shadowing program

No.	Item	Mean (SD)		Effect Size
		Pre-test	Post-test	
1	I know how an OR technician works with other members of the treatment team.	4.64 (±2.76)	7.92 (±1.81)	1.04***
2	I would like to be an OR technician.	4.46 (±2.08)	7.34 (±1.80)	0.98***
3	I am fully aware of OR technician’s the duties and responsibilities.	5.28 (±2.73)	7.90 (±1.43)	0.92***
4	I enjoy working as an OR technician.	5.58 (±2.74)	8.14 (±1.26)	1.02***
5	I intend to continue my education in OR academic field of study.	5.20 (±3.01)	8.50 (±1.18)	1.13***

*JMS* Job Motivation Survey, *SD* Standard Deviation

^*^P < 0.05

^**^P < 0.01

^***^P < 0.001

The mean (std. dev.) of pre-test and post-test total scores of JDS questionnaire were 57.78 (9.78) and 68.58 (5.02), respectively. Pre-test and post-test job diagnostic level was significantly different before and after the intervention (*P* < 0.001) (Table 2). The ES of the pre-test and post-test JDS’s total score was 0.95 showing strong impact of the program on enhancing freshmen’ job diagnostic (Table 1).

**Table 2:** Mean and standard deviation of Hackman and Oldham’s JDS’s items before and after the shadowing program

Item	Mean (SD)		Effect size
	Pre-test	Post-test	
**Job Characteristics: Skill Variety**			
The job require me to do many different things at work, using a variety of your skills and talents.	4.43 (±1.04)	4.86 (±0.35)	0.51**
The job requires me to use a number of complex or high-level skills.	3.60 (±1.16)	4.74 (±0.44)	1.08***
The job is quite simple and repetitive	3.66 (±1.30)	3.52 (±1.21)	0.07
**Job Characteristics: Task Identity**			
The job involves completing pieces of work that has an obvious beginning and end.	3.20 (±1.19)	4.12 (±0.82)	0.62***
The job provides me the chance to completely finish the pieces of work I begin.	3.06 (±1.15)	3.80 (±1.05)	0.52**
The job is arranged so that I do not have the chance to do an entire piece of work from beginning to end.	3.56 (±1.41)	3.30 (±1.14)	0.14
**Job Characteristics: Task Significance**			
The results of my work likely to significantly affect the lives or well-being of other people	3.58 (±1.35)	4.60 (±0.70)	0.64***
This job is one where a lot of other people can be affected by how well the work gets done.	3.38 (±1.46)	4.72 (±0.80)	0.80***
The job itself is not very significant and important in the broader scheme of things.	4.00 (±1.38)	3.52 (±1.35)	0.27
**Job Characteristics: Autonomy**			
The job gives me considerable opportunity for independence and freedom in how I do the work.	2.88 (±0.98)	3.56 (±0.99)	0.51**
My job permit me to decide on my own how to go about doing the work.	2.76 (±1.17)	3.90 (±0.97)	0.73***
The job denies me any chance to use my personal initiative or judgment in carrying out the work	3.12 (±1.30)	2.84 (±0.97)	0.16
**Job Characteristics: Feedback from the job itself**			
The actual work itself provide clues about how well you are doing—aside from any “feedback” coworkers or supervisors may provide.	3.34 (±1.20)	4.34 (±0.82)	0.71***
Just doing the work required by the job provides many chances for me to figure out how well I am doing.	3.14 (±1.17)	4.34 (±0.71)	0.92***
The job itself provides very few clues about whether or not I am performing well	3.42 (±1.09)	3.50 (±1.37)	0.04
**Feedback from agents**			
Other people (such as managers and colleagues) provide feedback about effectiveness (e.g., quality or quantity) of my job performance.	3.18 (±1.30)	4.26 (±0.82)	0.86***
**Interaction with colleague**			
In my job, I have to cooperate closely with other healthcare team members.	3.48 (±1.46)	4.74 (±0.72)	0.77***

*JDS* Job Diagnostic Survey, *SD* Standard Deviation

*P < 0.05

**P < 0.01

***P < 0.001

The Pearson correlation coefficient between the pre-test total scores of both JDS and JMS questionnaires was 0.64, which was statistically significant (*P* < 0.001). In addition, the post-test total scores of both JDS and JMS questionnaire was 0.18, which was not statistically significant (*P* = 0.2).

## Discussion

The quality of the medical education system can affect students’ job motivation and diagnostic. Decreased job motivation and academic failure as one of the major problems of the education system in countries causes a waste of many resources and training of unskilled graduate [40]. Studies conducted in Iran show that every year a large number of students due to dissatisfaction with the field of study, drop out of school or have low motivation to continue their education [41]. Educational institutions seek to promote and improve students’ motivation to future professions and use appropriate strategies to provide new and innovative educational opportunities [42]. By implementing the shadowing program in this study, we have provided the conditions for new OR students to be exposed to their future work environment in. Based our results, a shadowing program considerably enhanced students’ job motivation and job diagnostic.

Through the shadowing program, students’ job diagnostic and job motivation increased by facing the realities of the OR clinical environment. Liu et al. believed that shadowing programs helped the students in gaining knowledge to meet the requirements of future job and workplace as well as in enhancing students’ job motivation and later job satisfaction [43]. This study’s findings showed a broad shift in OR students’ motivation to future profession and in efforts to succeed in this profession. Significant increase in JDS scores indicated that students had a direct contact with OR environment and the OR surgery team (OR staff, surgeon and anesthesia) through shadowing program. Consequently, students achieved a clearer view of their academic field and a more positive outlook on their future profession and their role on the healthcare team. In line with previous studies [44], this study showed that enhancing job recognition leads to significant changes in job motivation. Since Iran’s university admission system is through the national entrance exam and students enter directly from high schools, many students do not have a clear idea of ​​their future job related to their academic field of study. Particularly, students are very unlikely to have such experiences before entering universities in Iran. Kass et al. reported high ratings on core dimensions of the job characteristics were significantly associated with a better educational experience, and a stronger intent to remain at the university [14]. Hence, implementing a 2-day shadowing program in the first months of entering the OR academic field can have positive outcomes in attitudes and motivation’ students towards their job as other studies [9, 32, 45].

Students’ understanding of inter-professional interactions was improved considering the increase in score of JDS’s interaction-with-colleagues item [31, 46, 47]. Since part of OR clinical education in OR-related professions interconnects with interpersonal relations and professional interactions, this program function in preparing a realistic mindset for students. Ruiz et al. stated that a shadowing program provided advanced knowledge of their job characteristics compared to those of other professions [48, 49].

Studies show that students who are in contact with the clinical environment during their education or who voluntarily worked in clinical settings before their academic education both have an improved perspective of their field of study and are more interested and satisfied with their academic education [50]. After the program, results indicated that OR profession was quite different from what students previously thought in various aspects, including the skill variety and task identity. In addition to providing opportunities for experiencing independence and freedom of action, students gained a clear understanding of an OR technician’ duties in accordance with the principles and guidelines. Students experiencing this program can make more informed decisions regarding continuing their academic education by knowing the clinical environment and future profession. They can also improve required professional skills through such programs [51]. This study’s finding is in line with those of Wang et al. [22]; this change does not cause despair and frustration in students, instead increases their job motivation and their interest in seriously pursue their academic education.

Before the intervention, JDS and JMS questionnaires’ total pre-test scores indicated a positive and strong correlation between job recognition and job motivation. However, total post-test scores after the intervention showed a positive yet weak correlation between job motivation and recognition. According to these findings, the more job recognition students have before clinical phase of attending OR, the higher their job motivation. However, after students’ early exposure to OR through the program, increasing job recognition is positively correlated with increasing job motivation. Nevertheless, this correlation is not strong, which can be due to students’ exposure to OR realities, OR work pressure, and OR environment’s stressful conditions.

The major contributions of this study are as follows: (i) Design and implementation of shadowing program for OR students, (ii) Evaluation of this program’ impact on increasing freshmen’s job diagnostic and motivation; (iii) Purposeful selection of the shadowers; and, (iv) The empowerment shadowers through holding orientation program. A limitation of this study is that the program’s implementation and maintenance can be challenging, especially in critical environments such as OR. Paramedical faculties may act differently in their teaching hospitals, management policies, or availability of administrative support, all of which may affect the implementation of this model elsewhere. Another limitation is that the program evaluation was conducted as a cross-sectional survey and the results of the intervention were measured over a short period of time. Therefore, it is suggested that future studies be designed longitudinally or by considering a longer follow-up period to consider the effect of time on the impact of the intervention, as well as future longitudinal studies on the impact of the intervention on other job variables, including responsibility should be designed.

The selection process of shadowers and monitoring proper implementation of the program are significant responsibilities to ensure that the guidelines are correctly followed and that the newcomer students (shadowees) are satisfied. Nevertheless, shadowing should be included in the formal curriculum of OR students before entering the clinical phase to help them plan their professional future. As future work, the impact of the shadowing program on the job motivation in the longer term should be evaluated though follow-up studies in various fields of study.

## Conclusion

In shadowing program, through students’ exposure to OR and familiarity with OR-related profession, they gain realistic insights on their academic field of study and their future profession. In shadowing program, using the opportunity to be present in the operating room environment and the real space of the clinic by accompanying senior students, it had an effect on students’ initial knowledge of the operating room and increase their motivation.

## Data Availability

The datasets generated and/or analysed during the current study are not publicly available due to the counter’s data sharing policy, but are available from the corresponding author on reasonable request.

## Abbreviations

OR: Operating Room
JDS: Job Diagnostic Survey
JMS: Job Motivation Survey
